# Kinetoplastid cell biology and genetics, from the 2020 British Society for Parasitology Trypanosomiasis and Leishmaniasis symposium, Granada, Spain

**DOI:** 10.1017/S0031182021000998

**Published:** 2021-09

**Authors:** Pegine B. Walrad, Mark. C. Field, Miguel Navarro, Derrick R. Robinson

**Affiliations:** 1 Department of Biology, York Biomedical Research Institute, University of York, YO10 5DD, UK; 2 School of Life Sciences, University of Dundee, Dundee, DD1 5EH, UK; 3 Institute of Parasitology, Biology Centre, Czech Academy of Sciences, 37005 Ceske Budejovice, Czech Republic; 4 Instituto de Parasitologia y Biomedicina “Lopez-Neyra” (IPBLN), Parque Tecnologico de Ciencias de la Salud, 18016 Granada, Spain; 5 CNRS, Microbiology Fundamental and Pathogenicity, UMR 5234, F-33000 Bordeaux, France

**Keywords:** Disease mechanisms, evolution, kinetoplastid, *Leishmania*, meeting report, mode of action, molecular parasitology, mRNA processing, nuclear functions, nuclear structure, proteolysis, *Trypanosoma*

## Abstract

The British Society for Parasitology (BSP) holds a biannual symposium devoted to the kinetoplastids, and seeks to cover the full gamut of research into these important organisms, and alternates with the Woods Hole Kinetoplastid Molecular Cell Biology meeting that serves a similar community. While normally embedded within the main BSP Spring meeting, on several occasions the symposium has enjoyed the opportunity of being hosted on mainland Europe. In 2020, the BSP was fortunate to spend some time in Granada in Spain, where a superb meeting with excellent science in a spectacular setting was overshadowed by news of an emerging novel coronavirus. In this editorial, we hope to have captured some of that excellent science and to highlight aspects of the many great papers and reviews in this special issue, as well as provide a few images from the meeting, which we hope for this who attended will bring back some fond memories.

## Graphical abstract

**Figure:**
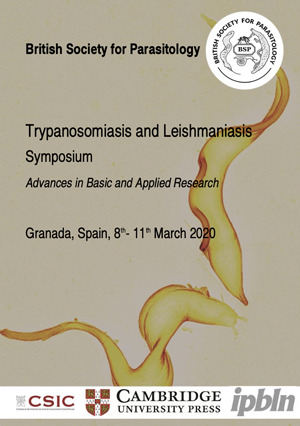

## Key findings

Many areas of cell biology are advancing apace in trypanosomiasis research, with many older conundrums yielding at last to understanding and better appreciation of the diversity within these organisms.

New tools, that include genome-/proteome-wide approaches and single cell RNA sequencing promise to open up understanding, with relevance to both basic biology and disease mechanisms.

International collaboration is increasing in our field, with the synergy that can arise from laboratory-based and field/clinic-based researchers working together now becoming prominent. This has improved diversity and representation both within molecular parasitology and at this meeting. Many members from partner organizations attended, including SOCEPA (Spain), DGP (Germany), SBPz (Brazil) and others.

Increased global research networks has both extended engagement and is actively promoted by The British Society for Parasitology. The symposium maintained the BSP's well-established reputation as a respected organizer of excellent and popular international meetings.

## Background

The British Society for Parasitology (BSP) Trypanosomiasis and Leishmaniasis Symposium; Advances in Basic and Applied Research held in Granada, Spain was unexpectedly both the sole BSP meeting of 2020 and the last time many of us have seen each other in person. The breaking coronavirus disease-2019 (COVID-19) pandemic prevented many from our community attending due to the real and rapidly growing health risk. Many of those that did attend returned home through desolate airports that would become all too familiar in the days and months that followed while some delegates were forced to quarantine. Despite these considerable obstacles, the symposium hosted scientists from over 20 countries and five continents (Table 1).

**Table 1.** Symposium attendees were from more than 50 institutes, 20 countries and five continents.

Country	Institutions represented
Argentina	University of San Martin
Australia	Flinders University and University of Technology Sydney
Belgium	University of Antwerp
Brazil	Fundação Oswaldo Cruz, University of Minas Geraïs, University of Rio de Janeiro and University of São Paulo
Colombia	University Nacional
Czech Republic	Charles University in Prague, University of Southern Bohemia and University of Ostrava.
France	University of Montpellier and Institut Pasteur
Germany	University of Heidelberg, University of Munich and University of Würzburg
Ghana	University of Accra, University of Ghana and University of Legon
Kenya	Kisii University
Nigeria	Zaria University
Malawi	Open University
Poland	University of Warsaw
Portugal	University of Lisbon
Saudi Arabia	King Faisal University
Spain	Universidad Granada and IPBLN-CSIC Granada
Sweden	University of Stockholm
Switzerland	University of Basel and University of Bern
Trinidad and Tobago	University of the West Indies
United Kingdom	University of Bristol, University of Cambridge, University of Dundee, University of Edinburgh, University of Glasgow, University of Huddersfield, University of Liverpool, Newcastle University, Nottingham University, University of Oxford, University of St. Andrews' and University of York
United States of America	Brown University, University of Georgia, George Washington University, Johns Hopkins University, University of Massachusetts, Northeastern University, Ohio State University, Rockefeller University and SUNY Buffalo,

While many colleagues were sorely missed as they had elected to not attend, their absence provided an unexpected silver lining and the symposium saw an unprecedented number of talks from early career researchers. The diversity amongst speakers' age, career stage, race and gender soared well above usual. There was an informal feel and air of excitement and opportunity *albeit* with a background of growing concern. Not only did the global situation spark debate, but it created an atmosphere where conversations were open and collaborations were instigated. Either despite or because of the huge crisis on the horizon, we enjoyed ourselves tremendously and toasted the tremendous weather, culture and science at Granada with close friends and colleagues. Here, we highlight some of the relevant advances from many excellent presentations and first-hand experience from the authors who did attend (Figs 1–4).

**Fig. 1.**
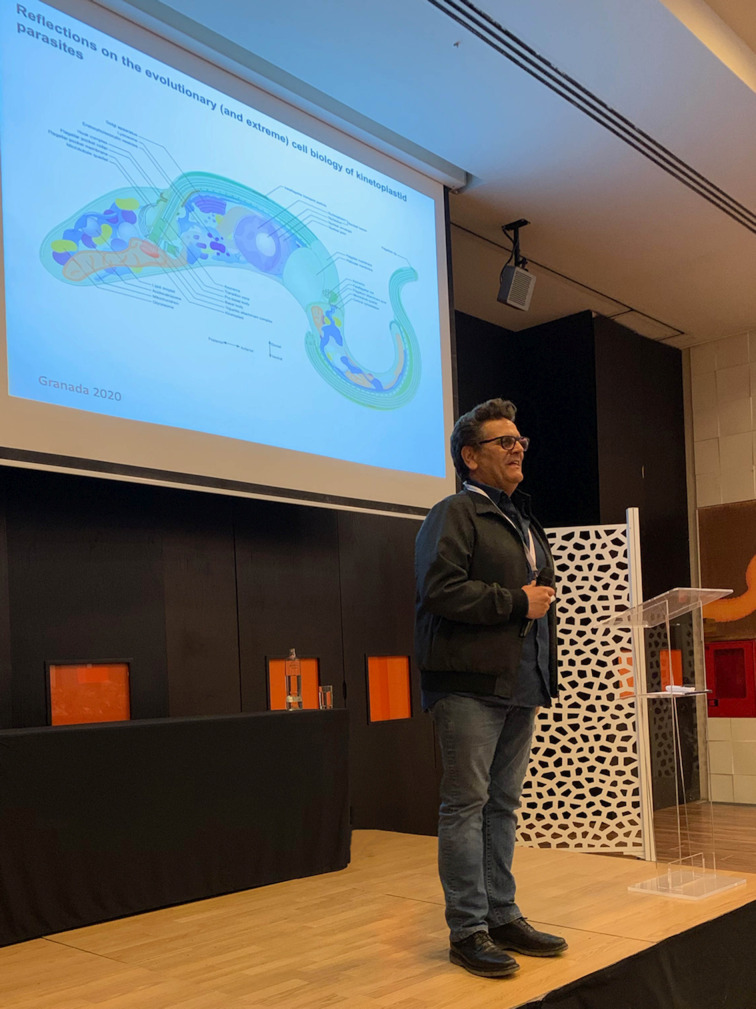
Miguel Navarro opens the meeting. Image © 2020 Mark C. Field, reproduced with permission.

**Fig. 2.**
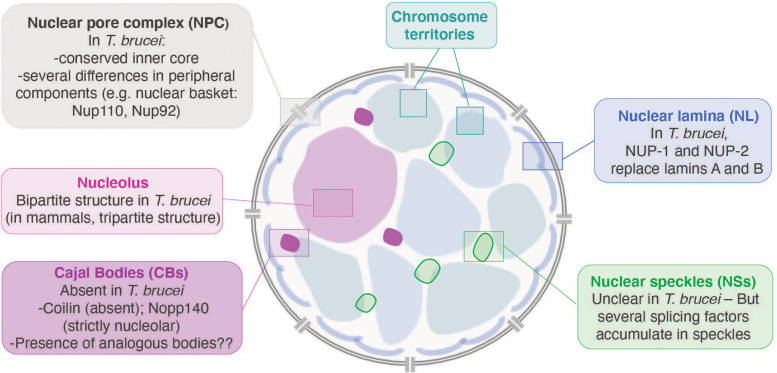
Nuclear organization and substructures in a generic cell. Boxes highlight features specific to trypanosomes. Modified from an illustration by Joanna Faria (Faria, 2021).

**Fig. 3.**
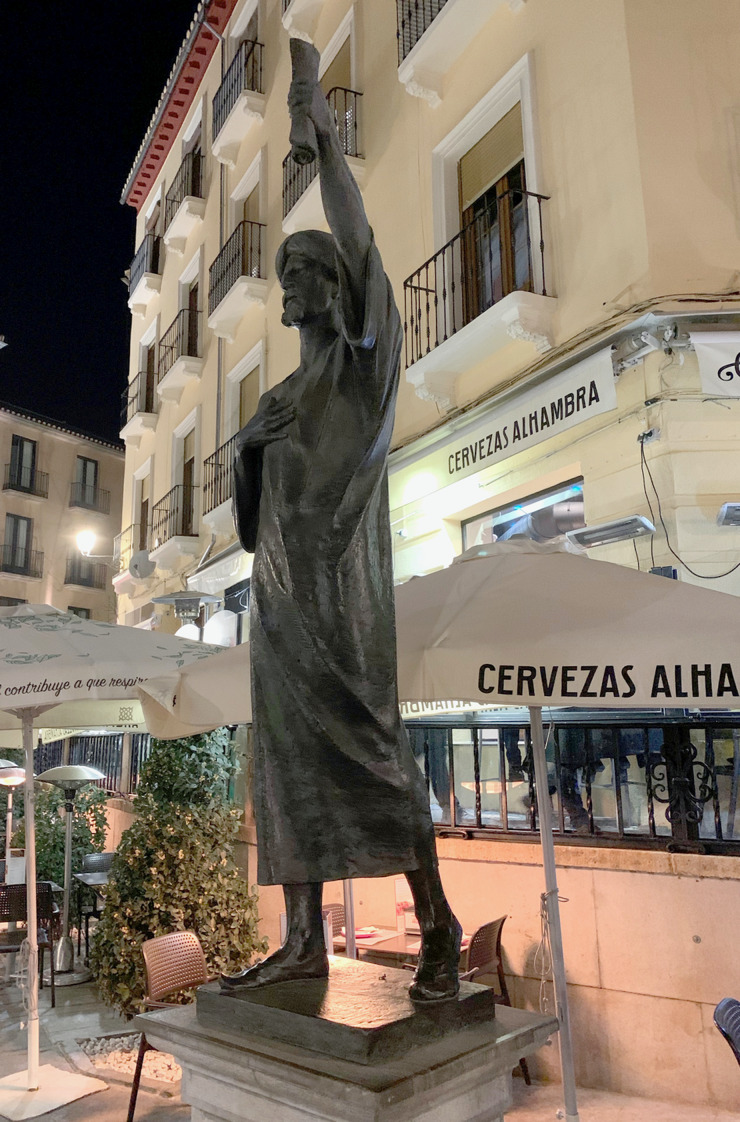
Statue of Yehuba Ibn Tibon, Calle Pavaneras, Granada, Spain. Ibn Tibon (1120 Granda – c1190 Marseille) was an important transzlator, particularly of medical and scientific works from Arabic into Latin and Hebrew, and is known as the father of translators. Statue donated to the city by Gutierre Ibn Tibón (a descendant) and designed by local sculptor Miguel Moreno. Image © 2020 Mark C. Field, reproduced with permission.

**Fig. 4.**
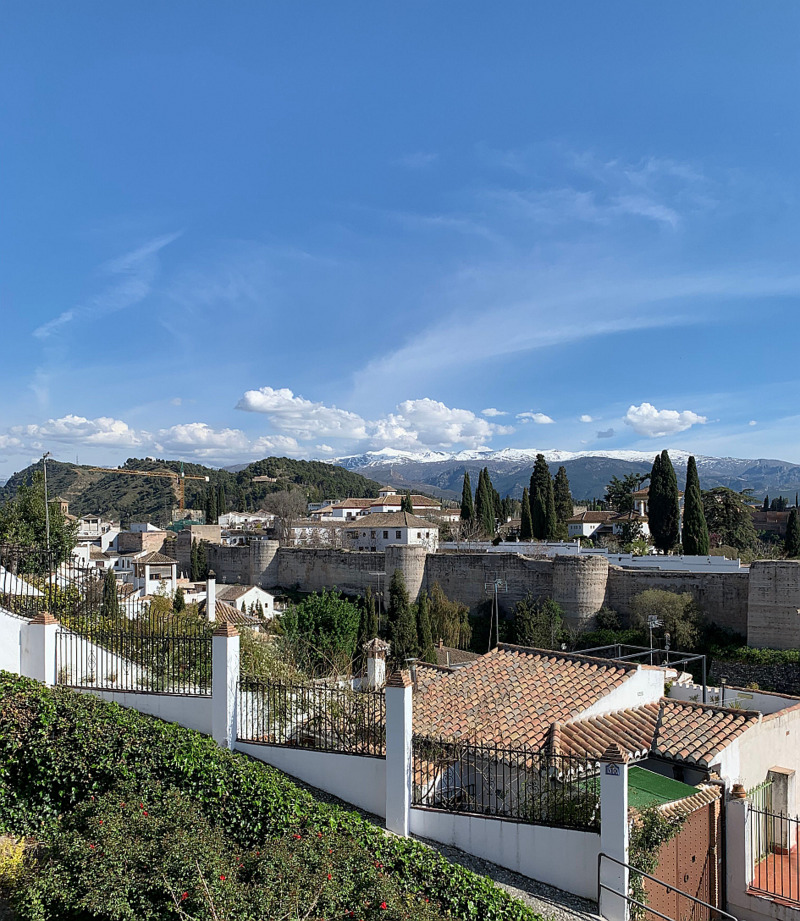
The Sierras. A view across part of the outskirts of Granada, across the city wall and onto snow-capped Sierra Nevada mountains. Image © 2020 Mark C. Field, reproduced with permission.

## New species and drug action

Trypanosomatids infect a wide variety of native Australian animals, including some endangered species. Thus, understanding the distribution of trypanosomes in Australian fauna is important not only from a conservation perspective but in understanding the ecology and biology of host−pathogen interactions. At least one lineage of Australian trypanosomes fall within the clade that contains the pathogen *Trypanosoma cruzi*, a parasite normally associated with South America, but which also contains African representatives such as *T. grayi*. John Ellis and his collaborators used phylogenetic analysis to show that the clade containing *T. cyclops* and a newly identified trypanosome from terrestrial leeches located around Sydney, in New South Wales, are very closely related, suggesting that they may be of the same species. Indeed, the genetic similarity and biogeography analysis permitted them to describe this leech parasite as a new subspecies of *T. cyclops* that they named *T. cyclops australiensis* (Ellis *et al*., 2021). Importantly, this highlights the historical connection between South East Asia and Australia within the paeleo-supercontinent Gondwanaland, and they hypothesize that evolution of the *T. cyclops* clade likely was underway before Gondwanaland broke up during the Jurassic period.

Two of the papers in this issue, which are a small snapshot of considerable activity within this area, address drug action. Firstly, TbAQP2 has been implicated in drug resistance in African trypanosomes and was identified by novel molecular approaches. In 2012 genome wide RNAi screens were used to identify pathways that facilitate anti-trypanosomal drug action and mechanisms involved in drug sensitivity and resistance. The article by Quintana and Field reviews the diversity, history and stability of TbAQP2 in terms of structure and function (Quintana and Field, 2021) and addresses the general question of the *T. brucei* protein stable as TbAQP2 possesses modified central core domains. TbAQP2 is the product of a trypanosome-specific duplication which has replaced the molecular NPA/NPA filter with NPS/NSA motif and the canonical aromatic/arginine (ar/R), while TbAQP2 is ubiquitylated and degraded *via* the lysosome. Finally, TbAQP2 is highly sensitive to mutation, which influences structure and can impair oligomerization leading to mis-localization and increased turnover highlighting possible mechanisms for petamadine resistance.

The second paper in this area examines melarsoprol, the arsenic-based drug used to treat late-stage trypanosomiasis (Larson *et al*., 2021). Although melarsoprol is chemically quite promiscuous and reacts with dithiol groups of many molecules, the key molecule here is trypanothione, a thiol important for maintaining the redox balance in trypanosomes and which is distinct to its human counterpart, glutathione, making it a novel and highly pursued drug target. Trypanothione reduces tryparedoxin which would normally deliver electrons to ribonucleotide reductase. Equally important is that reduced ribonucleotide reductase is required to produce dNTPs for DNA synthesis. Once taken in, melarsoprol is converted to melarsen oxide and binds tryparedoxin forming the adduct MelT, which in turn is an inhibitor for trypanothione reductase. Here Larson *et al*., demonstrate that eflornithine, fexinidazole and pentamidine cause significant increases in the proportion of cells in S and G_2_ phases which likely reflect delayed cytokinesis, whereas melarsoprol reduces S and G_2_ phase cell frequency, indicating a failure to enter mitosis. Overexpression of *γ*-glutamylcysteine synthetase, an enzyme representing the rate-limiting step in trypanothione biosynthesis, recovered cell cycle progression during melarsoprol treatment and this also correlates with an increase in DNA synthesis. This suggests that this pathway, and production of trypanothione, is critical for dNTP synthesis and hence melarsoprol sensitivity.

## Novel mechanisms in the trypanosome mitochondrion

The trypanosome mitochondrion is of considerable interest due to high divergence with most other organisms, including the presence of RNA-editing of mitochondrial genome encoded mRNAs and the recent description of a highly divergent mitoribosome in African trypanosomes. F-type ATP synthases couple ATP synthesis with proton translocation across biological membranes. Importantly, in some trypanosomes these complexes act in two ways. In the *T. brucei* insect form (procyclic), the mitochondrial genome encodes numerous oxidative phosphorylation-associated proteins, but some pathogenic trypanosome species can survive in the absence of a mitochondrial genome. The dyskinetoplastic species *T. evansi* and *T. equiperdum* are only viable as blood stage forms (BSF) and cannot develop in an insect vector, most likely because ATP production in insects requires oxidative phosphorylation and hence mitochondrial encoded genes. Changes to mitochondrial morphology between the procyclic form, which contains many cristae, and the BSF with few or none likely correlate with the ability to generate ATP (Guhara and Zíková 2021). Although an abundance of new data has been attained recently it is clear that we are far from understanding how the ATP synthase functions throughout the parasite life cycle and how this connects to alteration in cellular structures.

Complex I, the first component of the mitochondrial respiratory chain and hence of central importance, is nonessential in many kinetoplastid species, including the dyskinetoplastic *T. evansi* and *T. equiperdum* where it is absent. The molecular mass of complex I of dixenous parasites, i.e. those with both a vertebrate and invertebrate host, are remarkably larger than their metazoan counterparts, encoded by a combination of mitochondrial and nuclear genes and possess a considerable cohort of accessory proteins (Čermáková *et al*., 2021). As complex I of *T. brucei* is nonessential in both BSF and procyclic form, it is a possibility that the complex is modified or lost in other species. Significantly, complex II can act as the major entry site for electron flow through the respiratory chain. Complex I composition is highly variable across the trypanosomatids, and as shown in the paper here, in monoxenous parasites, i.e. those that have only insect hosts, there are novel proteins indicating significant diversity and likely accounting for differential sensitivity towards classic inhibitors, such as rotenone.

## Gene expression and nuclear organization

Control of gene expression in trypanosomes is mainly directed by post-transcriptional mechanisms regulating differentiation and survival. Premature or delayed differentiation can block life cycle progression and hence infection. Differentiation in *T. brucei* is induced by the activity of several key mRNA-binding proteins including, but not limited to, zinc-finger proteins (ZFPs) 1, 2 and 3 and RNA-binding proteins (RBPs) 6, 7 and 10. Using *T. cruzi* transcriptome data from cultured epimastigotes, cultured trypomastigotes and intracellular amastigotes Tavares and colleagues identify differentially expressed RBP-encoding genes (Tavares *et al*., 2020). They identify TcZH3H12, a zinc-finger RBP with a *T. brucei* orthologue that is upregulated in insect-infecting forms. The authors suggest a novel correlation between TcZH3H12 and PAD (proteins associated with differentiation) expression, the latter a cohort of proteins initially identified in African trypanosomes and, as the name suggests, with an expression profile associated with differentiating cells. They discuss the potential for an evolutionary conserved functional link between TcZH3H12 and PAD, which is of significant interest as a substantial amount is known for *T. brucei* and rather less for the other trypanosomatids which remain a health threat. Continuing this theme, Erben *et al*., present both functional data of three distinct RNA-binding proteins as well as an interesting classification method to compare transcripts associated with these and other RBPs (Erben *et al*., 2021). Such classification uses attributes including product function, transcript size, untranslated region length and stage expression. The authors seek to correlate mRNA length and RBP-binding motif consensus sequences and effectively categorize mRNPs in a meaningful way beyond a simple functional categorization of encoded proteins.

Expanding upon these data-driven RBP-centric articles are two reviews focusing upon differential aspects and pathways of RNA export. One explores the relative conservation of mRNA biogenesis, processing and export between trypanosomes and animals and fungi. This provides a comprehensive framework of great value to anyone teaching or embarking upon the field of protozoan gene regulation, as well as those with specific interests in protist evolution (Kramer, 2021). A complementary minireview by Paris on tRNA export examines conservation of a more discrete but equally vital process (Paris, 2021). Finally, Briggs and colleagues review the explosive impact of single cell transcriptomics (scRNASeq) upon gene-expression investigations, and their application to our field (Briggs *et al*., 2021). This paper highlights key contributions of this strategy to inform other systems with the potential to completely revise current knowledge and assumptions for gene-expression dynamics in parasite populations. Studies by these early career authors are pioneering this technique to a new audience of kinetoplastid researchers and provide new perspectives, likely challenging our understanding of trypanosomatid gene regulation and signalling.

Nuclear architecture, the positioning of genes and subnuclear compartmentalization are vital for regulating gene expression. A spectacular example was the identification of the expression site body (ESB) in African trypanosomes discovered by our meeting's host, Miguel Navarro. The ESB is a nuclear sub-compartment from which the single active variant surface glycoprotein (VSG) is transcribed. Faria discusses many novel and conserved processes within the trypanosome nucleus, emphasizing a cohort of highly conserved proteins that are involved in telomeric organization and hence silencing of VSG expression sites (Faria, 2021). Recent work implicates the arrangement of chromatin and the segregation of telomeric regions in trypanosomes, with RAP1, TRF, TIF2 and TelAP1 likely to be involved. Further, the ribonuclease acting at telomeric R-loops also plays significant roles in controlling VSG switching, and together with observations of the importance of histone modification, the VEX complex and a plethora of other factors is a challenge for the future. A complementary analysis reports on the composition of trypanosomatid telomere-associated protein complexes (Poláková *et al*., 2021). Comparative genomics indicates ~20 telomere-associated proteins are conserved across the kinetoplastids, *albeit* with several potential gains and losses. Significantly these alterations in gene repertoire are concentrated within several sub-lineages, with at least two new genes in trypanosomes expressing VSG, together with three new genes differentiating *Bodo saltans*, a free-living trypanosomatid relative, from all remaining kinetoplastids.

## Trafficking and targeting

There is considerable interest in kinetoplastid surface protein turnover as part of developmental programs, as well as for immune evasion. Hydrolase (mainly peptidase) activity within the endosomal systems of trypanosomes and Leishmania have been well documented, with the *proviso* that substrate/enzyme assignments remain few. A particularly interesting example of an endosomal resident is p67, an essential transmembrane domain glycoprotein mainly located at the lysosome. The canonical LAMP glycoproteins of metazoan cells are absent from kinetoplastids, and for many years it was thought that p67 represented a LAMP analogue. Extensive work from the Bangs laboratory has demonstrated that such a simplistic view is inaccurate, especially as the p67 polypeptide is extensively processed into two cleavage products, p32 and p42, that form a non-covalent complex in the lysosome. Recent work now makes two very important additions to this; firstly, that p67 has orthologs across eukaryotes, and that p67 is most likely a phospholipase B (PLB) relative (Koeller *et al*., 2020). PLB is capable of hydrolysis of fatty acids linked to both the *sn*-1 and *sn*-2 positions of glycerol, but the precise role that a PLB-like enzyme has in any organism remains to be reported. It is, of course, interesting to speculate that in trypanosomes this may be associated with turnover of GPI-anchored proteins and possible recycling of GPI-anchor constituents. With this indication of the p67 molecular function, a fuller understanding will likely be forthcoming soon.

The meeting also featured several notable talks discussing potential paradigm-shifting findings in *T. brucei* transcription, anti-kinetoplastid compound screens and novel factors implicated in *T. gambiense* serum resistance. Results from the Navarro lab suggest revision of our concept of *T. brucei* gene regulation is in order with the identification of candidate promoter of elements necessary and sufficient to drive reporter gene expression. Highlighting promising outcomes from the three anti-kinetoplastid compound boxes from GSK, Julio Martin (Biology Head, Kinetoplastid Unit, GSK) presented the latest advances on two candidates against visceral leishmaniasis that target a cyclin-dependent kinase and are currently in preclinical studies. A novel mannose-binding lectin that may contribute to *T. b. gambiense* ApoL1 sequestration and parasite survival in humans was described by Jean-Mathieu Bart. Finally, outstanding poster presentations went to PhD candidates Tania Bishola (DKFZ-ZMBH Alliance, Germany) and Malimba Lisulo (University of Edinburgh, UK), awarded for exploring 3′ UTR-dependent *trans*-regulation of RBP10 in *T. brucei* and for uncovering the roles of indigenous dogs in transmitting African trypanosomiasis in Zambia, respectively.

## Closing remarks and looking forward

In March 2020 and subsequent months there was much talk of ‘getting a vaccine for COVID’ but nothing tangible was available, despite previous viral epidemics in Asia and the Middle East. A year on there are multiple vaccines being used or tested, and in many countries (sadly not all) vaccination is underway. This strongly affirms the importance of basic and clinical research, much of which is attributable to efforts of biochemists, molecular biologists, parasitologists and other infectious disease workers and underscores the importance of international collaboration, cooperation and generosity. The meeting closed under the lengthening shadow of COVID-19, with news of mounting chaos, panic and disrupted travel. In our highly international community, we all felt this acutely and few predicted the immense challenges of the following year. Writing close to the anniversary of the first UK lockdown on 23^rd^ March 2020, this is a poignant reminder of those we have lost, but also of resilience in retaining connections, collaborations and advances in our science.
